# Dietary bamboo leaf flavonoids improve quality and microstructure of broiler meat by changing untargeted metabolome

**DOI:** 10.1186/s40104-023-00840-5

**Published:** 2023-04-06

**Authors:** Guangtian Cao, Huixian Wang, Yang Yu, Fei Tao, Huijuan Yang, Shenglan Yang, Ye Qian, Hui Li, Caimei Yang

**Affiliations:** 1 College of Standardisation, China Jiliang University, Hangzhou, 310018 People’s Republic of China; 2 Key Laboratory of Applied Technology On Green-Eco-Healthy Animal Husbandry of Zhejiang Province, Zhejiang Provincial Engineering Laboratory for Animal Health and Internet Technology, College of Animal Science and Technology, Zhejiang A & F University, Hangzhou, 311300 People’s Republic of China; 3 Zhejiang Vegamax Biotechnology Co., Ltd., Anji, 313300 People’s Republic of China

**Keywords:** Bamboo leaf flavonoid, Broiler, Meat quality, Metabolome, Protein secondary structure

## Abstract

**Background:**

Dietary bamboo leaf flavonoids (BLFs) are rarely used in poultry production, and it is unknown whether they influence meat texture profile, perceived color, or microstructure.

**Results:**

A total of 720 one-day-old Arbor Acres broilers were supplemented with a basal diet with 20 mg bacitracin/kg, 50 mg BLFs/kg, or 250 mg BLFs/kg or without additions. Data showed that the dietary BLFs significantly (*P* < 0.05) changed growth performance and the texture profile. In particular, BLFs increased birds’ average daily gain and average daily feed intake, decreased the feed:gain ratio and mortality rate, improved elasticity of breast meat, enhanced the gumminess of breast and leg meat, and decreased the hardness of breast meat. Moreover, a significant (*P* < 0.05) increase in redness (a*) and chroma (c*) of breast meat and c* and water-holding capacity of leg meat was found in BLF-supplemented broilers compared with control broilers. In addition, BLFs supplementation significantly decreased (*P* < 0.05) the β-sheet ratio and serum malondialdehyde and increased the β-turn ratio of protein secondary structure, superoxide dismutase, and glutathione peroxidase of breast meat and total antioxidant capacity and catalase of serum. Based on the analysis of untargeted metabolome, BLFs treatment considerably altered 14 metabolites of the breast meat, including flavonoids, amino acids, and organic acids, as well as phenolic and aromatic compounds.

**Conclusions:**

Dietary BLFs supplementation could play a beneficial role in improving meat quality and sensory color in the poultry industry by changing protein secondary structures and modulating metabolites.

## Background

Currently, promoting growth performance and optimizing product quality are the two main factors driving livestock production, in which the pressure increases over time [1]. Meat quality-related parameters, such as flavor, texture, color, and fatty acid composition, as well as potential bioactive compounds, have recently attracted consumers’ attention [2]. Improving these parameters is critical for researchers and has become more difficult because the inhibition of the use of antibiotics and hormone-based growth promoters in the poultry and swine industry, owing to antibiotic resistance, environmental pollution, and potential harmful influence on the health of consumers [3, 4]. Substitutes for antibiotics and hormone-based growth promoters with no adverse effects are necessary to enhance livestock growth performance and health; therefore, plant extract substances rich in antioxidants, mainly phenols, polyphenols, flavonoids, and amines, may be able to achieve this effect [5].

Polyphenols are plant-derived secondary metabolites that can modulate the sensory quality and nutritional value of foods. Many studies have revealed that polyphenols have beneficial roles as antioxidant and antibacterial agents in animals, foods, and humans [6–8]. Specifically, plant-derived polyphenols can enhance immune responses and improve meat quality, growth, and slaughter performance of broilers [9, 10], which these effects have also been observed in animals such as carp and lambs [11, 12]. Flavonoids, another type of polyphenols, are primarily known as color pigments, and their biological functions have only recently been investigated [13, 14]. Flavonoids contain many pharmacological substances that could improve livestock growth performance and product quality when added to the diet [15, 16]. There is a growing trend of using flavonoids to improve color, pH and oxidative stabilities, sensorial quality, and shelf-life and restrain microbial and pH-dependent deterioration of broiler meat [17].

Recently, bamboo has gained attention in the field owing to its nutritive and therapeutic potential and its important effects on food production, the pharmaceutical industry, and livestock production [5]. In China, the antioxidant compounds of bamboo leaves have been listed in “GB 2760–2014 National Food Safety Standard—Standard for Uses of Food Additives” [18], and can be added to meat products, beverages, edible oils, and fried foods. In addition, several studies have revealed that bamboo leaf flavonoids (BLFs) also have positive effects on growth performance, meat quality, antioxidant status, and cholesterol metabolism in broilers [19, 20]. However, very few studies have investigated the effects of BLFs on broiler meat quality. Based on an untargeted metabolome, the present study was conducted to investigate the influence of BLFs on the normal texture profile, sensory color, microstructure, and secondary protein structure.

## Methods

### Animal ethics

All the experimental procedures applied in this study were reviewed and approved by the Animal Care and Use Committee of Zhejiang A&F University. All procedures involving live bird handling, management, and health care followed the regulations for the use of laboratory animals for scientific purposes and were implemented as per the Ethics Committee of Zhejiang A&F University, Hangzhou, China (SYXKzhe 2021–013).

### Animal designation and materials

A total of 720 one-day-old male Arbor Acres broilers were randomly assigned to four treatment groups (six replicates with 30 birds per pen). The broilers were fed with a basal diet: control (CON, without any antibiotics or other additions), supplemented with antibiotics (ANT, 20 mg bacitracin/kg), supplemented with a low amount of bamboo leaf flavonoids (BLFL, 50 mg BLFs/kg), or supplemented with a high amount of bamboo leaf flavonoids (BLFH, 250 mg BLFs/kg). All broilers had free access to food and water, and the experiment lasted for 42 d. At the beginning of the study, the room temperature was set at 35 °C and gradually decreased to 29 °C by 2 °C reduction/week. The basic diet composition and nutritional contents followed the National Research Council [21] recommendations are presented in Table 1. The nutritional value of the diets was determined using association of official analytical chemists procedures as described in the previous study [22].

**Table 1:** Composition and nutrient levels of the basal experimental diet (air-dry basis)

Items	Ages, d	
	1–21	22–42
Ingredients, %		
Corn	56.33	57.4
Soybean meal	24.5	19
Extruded soybean	5	4
Corn distillers dried grains with solubles	5	8
Corn gluten meal	2	3
Soybean oil	1.2	4.3
Limestone	1.3	1.3
Fermented soybean meal	1.67	0
Premix^a^^,^^b^	3	3
Total, %	100.00	100.00
Nutrient levels		
Metabolizable energy, MJ/kg	12.34	13.17
Crude protein, %	20.6	18.6
Crude fat, %	4.9	8.0
Lysine, %	1.17	0.99
Methionine + Cysteine, %	1.45	1.23
Threonine + Tryptophan, %	1.13	0.95
Calcium, %	0.88	0.79
Total phosphorus, %	0.64	0.56

^a^Minimal vitamin levels per kg of diets: vitamin A (retinyl acetate), 1500 IU; cholecalciferol, 200 IU; vitamin E (*DL*-α-tocopheryl acetate), 10 IU; riboflavin, 3.5 mg; pantothenic acid, 10 mg; niacin, 30 mg; cobalamin, 10 μg; choline chloride, 1,000 mg; biotin, 0.15 mg; folic acid, 0.5 mg; thiamine 1.5 mg; pyridoxine 3.0 mg

^b^Minimal mineral levels per kg of diet: Fe 80.00 mg; Cu 8.00 mg; Mn 60.00 mg; Zn 40.00 mg; I 0.18 mg; Se 0.15 mg

At the end of the trial, 24 broilers (one bird per replicate) were selected and slaughtered for further sample collection. Left breast and leg meat were obtained and stored at –80 °C prior to investigating meat quality, secondary structure of the protein, and untargeted metabolome detection. Moreover, crosscut muscle samples were fixed in 3% glutaraldehyde at 25 ℃ for microstructural analysis.

Similar to our previous study, the bamboo (*Dendrocalamus membranaceus*) leaf flavonoids were composed of 24.2% flavonoids, 12.5% lactones, and 15.6% phenolic acids [23], which were provided by Zhejiang Vegamax Biotechnology Co., Ltd. (Anji, China) and directly added to the basal diet. Moreover, the major compositions of BLFs are chlorogenic acid (21.33 mg/100 g), caffeic acid (1.85 mg/100 g), orientin (34.66 mg/100 g), isoorientin (34.66 mg/100 g), *p*-coumaric acid (26.69 mg/100 g), vitexin (148.42 mg/100 g), and fumalic acid (265.03 mg/100 g).

### Meat texture profile analysis

The texture measurements were performed using a Rapid TA + texture analyzer (Shanghai Tengba Instrument Technology Co., Ltd., Shanghai, P.R. China) with a spherical probe to reach 75% compression. The analyzer parameters were as follows: speed 1 mm/s, trigger force 5 N, and probe time 2 s.

### Meat sensory color

The sensory color of the meat samples was measured according to the CIE L*a*b* scale coordinates, including luminosity (L*), redness (a*), yellowness (b*), and chroma (c*). The values were analyzed using an SR-68 computer colorimeter (Shenzhen Threenh Technology Co., Ltd., Guangdong, P.R. China) and recorded at the central position of the sample meat at 15 ± 2 ℃.

### Meat pH

The pH of leg and breast meat was determined using an MP511 Lab pH meter (Shanghai San-Xin Instrumentation Inc., Shanghai, P.R. China) at 15 ± 5 ℃.

### Meat water-holding activity

Similar to Wu et al. [24], after slaughtering for 24 h, water activity (Aw) values of the breast and leg meat were measured using an HD-5 water activity meter (Wuxi Huake Instrument Co., Ltd., Jiangsu, P.R. China) at 10 ± 2 ℃.

### Meat microstructure

The muscle samples were stored in glutaraldehyde, post-fixed in 1% osmic acid, and dehydrated in gradient ethanol. Then, the samples were embedded in Epon-Araldite, cut into slices using Leica A-1170 (Leica DM IL, Leica, Germany), and stained with 2% uranium acetate and lead citrate. Ultrastructural images were obtained using a transmission electron microscope (Model H-7650, Hitachi, Japan) at an accelerating voltage of 15 kV and 7000 × magnification.

### Protein secondary structure of meat

Fourier transform infrared analysis spectra obtained from 400 to 4,000 cm^–1^ on silicon wafers were recorded using a Niocolet Fourier Transform (Thermo Fisher Scientific, Waltham, MA, USA). After 32 h of vacuum freeze drying, the broilers’ breast-meat samples were frozen at –40 ℃ for 12 h. The spectra of the samples were recorded as 512 scans, the resolution of the instrument was 4 cm^−1^, and the measurements were carried out at 25 ℃. The percentage of each secondary structure was calculated using Omnic 8.2.387 software (Thermo Fisher Scientific) and PeakFit 4.12 (Systat Software, San Jose, CA, USA). The overlapping bands in the deconvolved amide I region were resolved using a multipeak program with a Gaussian function to calculate the peak areas.

### Serum and breast meat antioxidative capacity

The antioxidative capacity of serum and breast meat, namely total antioxidant capacity (T-AOC), superoxide dismutase (SOD), malondialdehyde (MDA), catalase, and glutathione peroxidase (GSH-Px), were spectrophotometrically measured using commercial kits (Nanjing Jiancheng Institute of Biotechnology, Nanjing, P.R. China), according to the manufacturer’s instructions.

### Ultra high-performance liquid chromatography time of flight mass spectrometry for meat metabolome

In total, 50 mg of meat obtained from the breast and leg of broilers was used for untargeted metabolite extraction, and 24 biological samples were tested. For each meat sample, 400 μL of a precooled methanol–acetonitrile (1:1, v/v) mixture was added and homogenized using a TGrinder electronic tissue burnisher (Tiangen Biotech Co., Ltd., Beijing, China). The mixtures were ultrasonically extracted for 10 min and then stored overnight at –80 °C. Immediately, the mixtures were centrifuged at 3,000 × *g* at 4 °C for 15 min (Centrifuge 5424R, Eppendorf, Germany). Finally, the supernatant was combined with 100 µL methanol/acetonitrile (1:1, v/v) and ultrasonicated for 12 min. After another centrifugation (10,000 × *g*, 15 min, 4 °C), the supernatant was removed from the injection bottles for the final metabolite analysis. The Agilent 6545 Q-TOF/MS system (Agilent Technologies, Santa Clara, CA, USA) and UHPLC system with an Agilent Zorbox Eclipse Plus C-18 Column (2.1 mm × 100 mm, 1.8 mm; Waters Corp., Milford, MA, USA) were used for metabolite detection in the samples. The negative ionization mode was used for further processing, and the MS conditions were consistent with those used in our previous study [23].

### Statistical analysis

All data were analyzed using a one-way analysis of variance and Tukey’s pairwise-test using IBM SPSS 173 Statistics for Windows, version 21.0 (IBM Corp., Armonk, NY, USA) and GraphPad Prism software version 8.0.2 (GraphPad Software Inc., San Diego, CA, USA). Standardized metabolic data were imported into the SIMCA-P + 11.0 software package (Umetrics, Basel, Switzerland) for multivariate analysis. Principal component analysis and partial least squares discriminant analysis were used to evaluate the data. After orthogonal partial least squares discriminant analysis (OPLS-DA), the distinguished metabolites within all treatment groups were identified. The results are presented as mean ± standard deviation, and differences were regarded as significant at *P* < 0.05.

## Results

### Effects of BLFs on the growth performance of broilers

Table 2 showed that BLFs supplementation significantly improved (*P* < 0.01) the average daily gain (ADG) and average daily feed intake (ADFI) of broilers than CON treatment from d 1 to 42. Although no significant difference was observed in the BLF and antibiotic administration, these treatment significantly decreased (*P* < 0.01) the feed:gain ratio (F:G) and mortality rate of birds compared with the CON treatment.

**Table 2:** Effects of BLFs supplementation on growth performance of broilers

Item	Stage	CON	ANT	BLFL	BLFH	SEM	*P*-value
ADG, g/d	d 1–21	41.0 ± 1.39	38.4 ± 2.97	39.8 ± 2.42	38.3 ± 1.97	2.39	0.159
	d 21–42	91.7 ± 7.60^b^	100.5 ± 6.36^a^	103.3 ± 8.23^a^	103.2 ± 6.79^a^	8.35	0.037
	d 1–42	80.4 ± 4.28^b^	97.6 ± 4.74^a^	98.5 ± 2.35^a^	97.7 ± 5.29^a^	8.75	< 0.001
ADFI, g/d	d 1–42	135.1 ± 4.16^b^	142.2 ± 0.92^a^	147.2 ± 5.12^a^	147.4 ± 7.95^a^	7.03	0.005
F:G	d 1–42	1.64 ± 0.04^a^	1.54 ± 0.04^b^	1.56 ± 0.04^b^	1.55 ± 0.04^b^	0.05	0.001
Mortality rate, %	d 1–42	3.01 ± 0.63^a^	1.50 ± 0.22^b^	0.83 ± 0.09^b^	1.00 ± 0.09^b^	0.29	0.003

*ADG* Average daily gain, *ADFI* Average daily feed intake, *F:G* Feed:gain ratio. All measurements were expressed as Mean ± SD (*n* = 6). ^a,b^Means with different letters in the same rows are different significantly (*P* < 0.05). Birds supplemented with basal diet (CON), supplemented with 20 mg bacitracin/kg (ANT), supplemented with 50 mg BLFs/kg (BLFL) or supplemented with 250 mg BLFs/kg (BLFH)

### Effects of BLFs on carcass yield of broilers

As shown in Table 3, dietary BLFH supplementation significantly increased (*P* < 0.01) the leg meat percentage of broilers compared to CON and ANT supplementation, even though there was no significant difference in carcass yield between CON and BLF treatment. In addition, the breast percentage of BLFH birds was higher (*P* = 0.088) than that of CON birds.

**Table 3:** Effects of BLFs supplementation on carcass yield of broilers

Item	CON	ANT	BLFL	BLFH	SEM	*P*-value
Carcass yield, %	87.0 ± 1.56	89.1 ± 1.35	88.8 ± 1.82	88.6 ± 1.70	1.74	0.072
Breast meat percentage, %	16.6 ± 0.53	17.1 ± 0.89	17.3 ± 0.45	17.4 ± 0.69	0.70	0.088
Leg meat percentage, %	13.1 ± 0.53^b^	13.2 ± 0.54^b^	13.5 ± 0.64^ab^	14.2 ± 0.62^a^	0.72	0.003

All measurements were expressed as Mean ± SD (*n* = 6). ^a,b^Means with different letters in the same rows are different significantly (*P* < 0.05). Birds supplemented with basal diet (CON), supplemented with 20 mg bacitracin/kg (ANT), supplemented with 50 mg BLFs/kg (BLFL) or supplemented with 250 mg BLFs/kg (BLFH)

### Effects of BLFs on meat texture profile of broilers

#### Brittleness

Neither BLF nor ANT treatment influenced the brittleness of the leg and breast meat compared to that of the CON group (*P* > 0.05), although BLFL and BLFH increased the brittleness compared to that of the CON and ANT groups (Tables 4 and 5).

**Table 4:** Effects of BLFs supplementation on texture profile of broilers’ leg meat

Item	CON	ANT	BLFL	BLFH	SEM	*P*-value
Brittleness	179.7 ± 46.00	183.5 ± 42.87	213.1 ± 34.10	205.7 ± 34.70	40.44	0.275
Elasticity	0.30 ± 0.03	0.33 ± 0.04	0.32 ± 0.04	0.33 ± 0.04	0.04	0.332
Resilience	0.14 ± 0.02^b^	0.19 ± 0.04^a^	0.19 ± 0.03^a^	0.20 ± 0.03^a^	0.04	0.007
Gumminess	52.13 ± 9.10^c^	75.2 ± 12.56^b^	86.8 ± 12.58^ab^	93.7 ± 12.59^a^	19.61	< 0.001
Chewiness	43.68 ± 7.36^b^	49.2 ± 6.68^ab^	47.0 ± 7.22^ab^	52.1 ± 9.41^a^	7.90	0.210
Cohesiveness	0.26 ± 0.03	0.27 ± 0.05	0.27 ± 0.04	0.26 ± 0.03	0.04	0.744
Hardness	204.9 ± 32.96^a^	210.6 ± 29.47^a^	159.9 ± 53.64^b^	174.5 ± 32.89^ab^	42.30	0.039

All measurements were expressed as Mean ± SD (*n* = 6). ^a,b^Means with different letters in the same rows are different significantly (*P* < 0.05). Birds supplemented with basal diet (CON), supplemented with 20 mg bacitracin/kg (ANT), supplemented with 50 mg BLFs/kg (BLFL) or supplemented with 250 mg BLFs/kg (BLFH)

**Table 5:** Effects of BLFs supplementation on texture profile of broilers’ breast meat

Item	CON	ANT	BLFL	BLFH	SEM	*P*-value
Brittleness	207.3 ± 42.86	226.8 ± 48.46	201.1 ± 45.08	214.9 ± 30.65	30.65	0.654
Elasticity	0.26 ± 0.02^b^	0.29 ± 0.04^b^	0.30 ± 0.03^a^	0.30 ± 0.03^a^	0.03	0.031
Resilience	0.14 ± 0.53^b^	0.15 ± 0.54^ab^	0.17 ± 0.64^ab^	0.19 ± 0.62^a^	0.03	0.014
Gumminess	47.13 ± 7.82^b^	60.9 ± 14.55^ab^	81.1 ± 8.81^a^	80.2 ± 13.86^a^	18.18	< 0.001
Chewiness	34.85 ± 4.88^b^	47.48 ± 9.33^a^	42.4 ± 8.70^ab^	48.3 ± 12.18^a^	10.25	0.025
Cohesiveness	0.26 ± 0.03	0.27 ± 0.05	0.27 ± 0.04	0.26 ± 0.03	0.03	0.744
Hardness	243.3 ± 53.0^a^	182.7 ± 25.4^b^	156.2 ± 37.1^b^	177.1 ± 39.5^b^	50.33	0.001

All measurements were expressed as Mean ± SD (*n* = 6). ^a,b^Means with different letters in the same rows are different significantly (*P* < 0.05). Birds supplemented with basal diet (CON), supplemented with 20 mg bacitracin/kg (ANT), supplemented with 50 mg BLFs/kg (BLFL) or supplemented with 250 mg BLFs/kg (BLFH)

#### Chewiness

Compared to that of the CON group, dietary BLFs supplementation increased the chewiness of broiler breast and leg meat, and a significant difference was observed only in the BLFH treatment group (*P* < 0.05; Tables 4 and 5). Moreover, ANT treatment significantly increased the chewiness of broiler breast meat compared to the CON treatment (*P* < 0.05).

#### Cohesiveness

Feeding low amount of BLFs significantly decreased the cohesiveness of broiler leg meat compared to the CON treatment (*P* < 0.05; Table 4). Meanwhile, both BLFL and BLFH broilers had significantly lower cohesiveness of leg meat than ANT birds (*P* < 0.01, *P* < 0.05). Feeding with antibiotics or BLFs did not affect the cohesiveness of breast meat in comparison with that of the CON group (Table 5).

#### Elasticity

Both BLFL and BLFH treatments improved broiler breast and leg meat elasticity in comparison with the CON treatment (Tables 4 and 5), while significant differences were only observed in breast meat (*P* < 0.05).

#### Gumminess

BLFs supplementation remarkably enhanced (*P* < 0.001) the gumminess of bird breast and leg meat compared to the CON treatment (Tables 4 and 5). Moreover, BLFH treatment significantly increased the gumminess of leg meat compared with that of the ANT birds (*P* < 0.01). In addition, either BLFL or BLFH birds had a higher gumminess of breast meat in comparison with that of the ANT birds (*P* < 0.01, *P* < 0.05).

#### Hardness

BLFL feeding significantly decreased the hardness of the leg muscles of birds compared to the CON and ANT treatments (*P* < 0.05; Table 4). BLFH also decreased (*P* = 0.071) the hardness of leg muscles compared to the ANT supplementation. Both ANT and BLF supplementation significantly decreased the hardness of the breast meat of birds in comparison with the CON treatment (*P* < 0.05; Table 5).

#### Resilience

Feeding ANT, BLFL, and BLFH significantly increased the resilience of leg meat compared to the CON supplementation (*P* < 0.05; Table 4). Moreover, BLFL and BLFH supplementation enhanced the resilience of breast meat compared to the CON treatment (*P* = 0.085 and *P* < 0.05, respectively; Table 5).

### Effects of BLFs on Aw and pH of broilers’ meat

There was no significant influence on the Aw of breast meat when fed with ANT or BLFs compared with CON (Table 6). Meanwhile, BLFL, BLFH, and CON treatment dramatically increased the Aw of leg meat compared with the ANT treatment (*P* < 0.01; Table 6). Neither BLF nor ANT supplementation affected the pH of breast and leg meat compared to the CON treatment (*P* > 0.05; Table 6).

**Table 6:** Effects of BLFs on Aw and pH of broilers’ meat

Item	CON	ANT	BLFL	BLFH	SEM	*P*-value
Breast meat pH	6.10 ± 0.13	6.14 ± 0.12	6.07 ± 0.15	6.14 ± 0.96	0.12	0.687
Breast meat Aw	0.97 ± 0.01	0.98 ± 0.01	0.98 ± 0.01	0.98 ± 0.01	0.01	0.827
Leg meat pH	6.33 ± 0.12	6.24 ± 0.06	6.26 ± 0.12	6.28 ± 0.08	0.10	0.296
Leg meat Aw	0.98 ± 0.01^a^	0.97 ± 0.01^b^	0.98 ± 0.01^a^	0.98 ± 0.01^a^	0.01	0.001

*Aw* Water activity. All measurements were expressed as Mean ± SD (*n* = 6). ^a,b^Means with different letters in the same rows are different significantly (*P* < 0.05). Birds supplemented with basal diet (CON), supplemented with 20 mg bacitracin/kg (ANT), supplemented with 50 mg BLFs/kg (BLFL) or supplemented with 250 mg BLFs/kg (BLFH)

### Effects of BLFs on the sensory color of broilers’ meat

There was no significant difference in L*, a*, and b* of sensory color of broilers’ leg meat among the treatment groups (Table 7). Supplementation with BLFs dramatically increased (*P* < 0.05) the c* value of leg meat color compared to CON and ANT supplementation. Although BLFs feeding did not affect (*P* > 0.05) the L* value of the birds’ breast meat compared with CON treatment (Table 8), it significantly increased a* and c* values. No significant differences were found in the values of leg and breast meat between the two BLF treatment groups.

**Table 7:** Effects of BLFs supplementation on broilers’ leg meat colour

Item	L*	a*	b*	c*
CON	51.02 ± 1.23	5.28 ± 0.75	10.76 ± 1.26	13.05 ± 0.86^b^
ANT	51.51 ± 1.22	5.11 ± 0.09	9.96 ± 0.43	14.49 ± 0.59^b^
BLFL	52.58 ± 1.48	5.31 ± 0.13	10.61 ± 0.71	16.00 ± 0.71^a^
BLFH	55.41 ± 1.64	5.83 ± 0.12	11.31 ± 0.37	15.88 ± 0.52^a^
SEM	0.595	0.309	0.383	0.388
*P*-value	0.484	0.367	0.479	0.015

All measurements were expressed as Mean ± SD (*n* = 6). ^a,b^Means with different letters in the same column are different significantly (*P* < 0.05). Birds supplemented with basal diet (CON), supplemented with 20 mg bacitracin/kg (ANT), supplemented with 50 mg BLFs/kg (BLFL) or supplemented with 250 mg BLFs/kg (BLFH)

L* Luminosity, a* Redness, b* Yellowness, c* Chroma

**Table 8:** Effects of BLFs supplementation on broilers’ breast meat colour

Item	L*	a*	b*	c*
CON	51.73 ± 1.21	5.25 ± 0.23^b^	8.65 ± 0.62^b^	10.03 ± 0.45^b^
ANT	53.95 ± 1.87	5.23 ± 0.35^b^	9.35 ± 0.65^b^	11.91 ± 0.73^ab^
BLFL	53.08 ± 1.64	6.60 ± 0.31^a^	10.06 ± 0.58^ab^	12.03 ± 0.61^a^
BLFH	53.60 ± 2.58	6.90 ± 0.26^a^	11.98 ± 0.63^a^	12.71 ± 0.85^a^
SEM	0.910	0.172	0.369	0.398
*P*-value	0.850	0.007	0.005	0.017

All measurements were expressed as Mean ± SD (*n* = 6). ^a,b^Means with different letters in the same column are different significantly (*P* < 0.05). Birds supplemented with basal diet (CON), supplemented with 20 mg bacitracin/kg (ANT), supplemented with 50 mg BLFs/kg (BLFL) supplemented with 250 mg BLFs/kg (BLFH)

L* Luminosity, a* Redness, b* Yellowness, c* Chroma

### Effects of BLFs on the microstructure of broilers’ breast meat

**Fig. 1:**
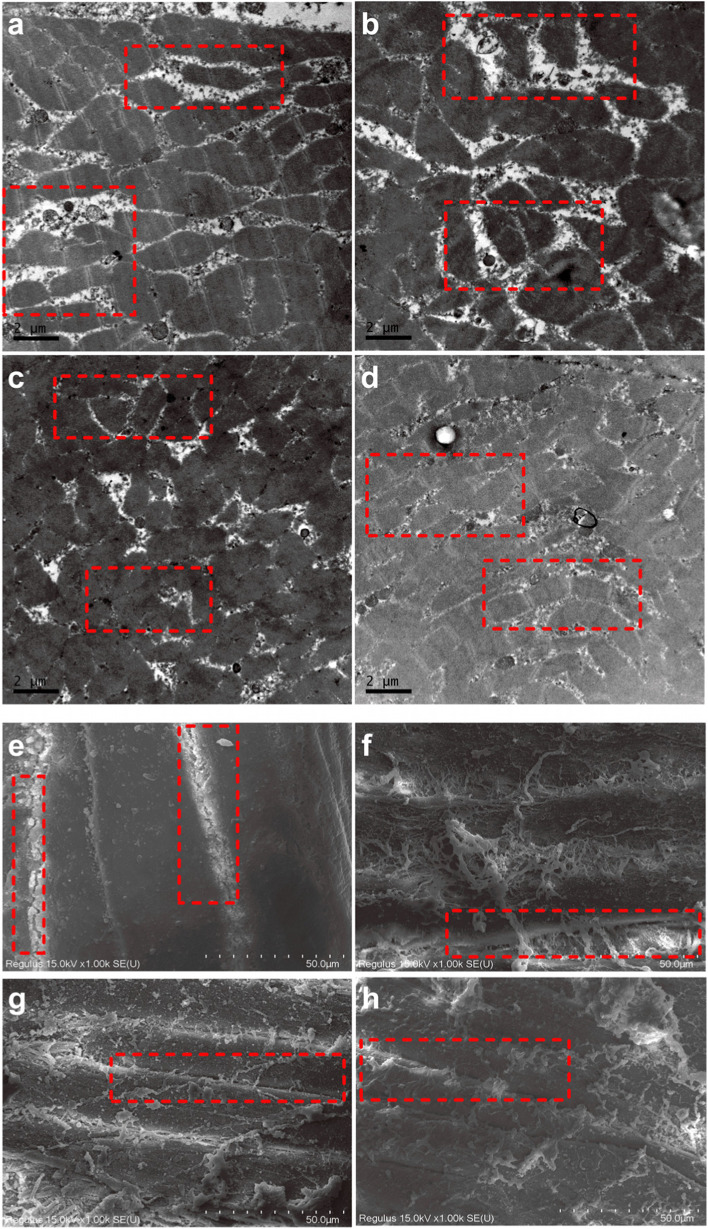
Effects of BLF on the microstructure of broilers’ breast meat. Note: **a**–**b** represents transection diagram of CON, ANT, BLFL and BLFH treatment, **e**–**h** represents slitting diagram of CON, ANT, BLFL and BLFH treatment, respectively (7000 ×). Birds supplemented with basal diet (CON), supplemented with 20 mg bacitracin/kg (ANT), supplemented with 50 mg BLFs/kg (BLFL), supplemented with 250 mg BLFs/kg (BLFH)

The breast microstructure marked by red boxes indicated that the BLF birds had less intramuscular fat than the CON and ANT birds, which the BLFH birds had the least intramuscular fat (Fig. 1a–d). Compared with that in the CON and ANT groups, intact myofibrils with distinct A-bands, I-bands, and Z-lines marked by red boxes were more clearly visible in the pectoral muscle of BLFL and BLFH groups (Fig. 1e–h). In addition, the breast muscle fibers in the BLF-treated birds were arranged in a more orderly manner, and there was less connective tissue present.

### Effects of BLFs on protein secondary structure of broilers’ breast meat

Data showed that BLF supplementation significantly decreased the β-sheet ratio and increased the β-turn ratio compared to the CON treatment (*P* > 0.05; Table 9). No significant difference was found in the ratio of α-helices and random coils among all treatment groups.

**Table 9:** Effects of BLFs supplementation on the secondary structures of broilers’ breast meat protein

Item	β-sheet, %	α-helix, %	β-turn, %	Random coil, %
CON	44.31 ± 1.245^a^	2.21 ± 0.836	52.41 ± 0.981^b^	1.07 ± 0.523
ANT	44.01 ± 0.831^a^	2.73 ± 0.586	51.87 ± 0.819^c^	1.39 ± 0.597
BLFL	41.59 ± 1.658^b^	1.95 ± 0.963	55.35 ± 1.810^a^	1.11 ± 0.506
BLFH	41.39 ± 1.036^b^	2.78 ± 0.276	54.76 ± 0.961^a^	1.08 ± 0.536
SEM	1.797	0.768	1.897	0.532
*P*-value	0.001	0.073	0.001	0.601

All measurements were expressed as Mean ± SD (*n* = 6). ^a,b^Means with different letters in the same column are different significantly (*P* < 0.05). Birds supplemented with basal diet (CON), supplemented with 20 mg bacitracin/kg (ANT), supplemented with 50 mg BLFs/kg (BLFL) or supplemented with 250 mg BLFs/kg (BLFH)

### Effects of BLFs on the antioxidative capacity of broilers’ serum and breast meat

No significant difference was noted in SOD and GSH-Px contents in broilers’ serum among the treatment groups (Table 10). The supplementation of BLFs significantly increased (*P* < 0.05) the content of serum total antioxidant capacity and catalase, whereas BLFH significantly decreased (*P* < 0.05) serum MDA content. Interestingly, high SOD and GSH-Px contents in breast meat were found in BLF-supplemented broilers.

**Table 10:** Effects of BLFs on antioxidative capacity of broilers’ serum and breast meat

Item		CON	ANT	BLFL	BLFH	SEM	*P*-value
Serum	T-AOC	12.7 ± 1.30^b^	13.2 ± 0.90^ab^	14.8 ± 1.38^a^	14.7 ± 0.79^a^	1.41	0.110
	MDA	10.3 ± 0.45^a^	9.8 ± 0.95^a^	9.1 ± 1.26^ab^	7.8 ± 0.90^b^	1.30	0.178
	SOD	87.3 ± 15.17	94.7 ± 12.23	96.2 ± 11.60	98.3 ± 10.93	12.47	0.448
	GSH-Px	9.9 ± 0.56	10.1 ± 0.52	10.5 ± 0.82	10.5 ± 0.91	0.74	0.402
	CAT	6.2 ± 0.58^b^	7.1 ± 1.07^a^	7.2 ± 0.89^a^	7.2 ± 0.72^a^	0.89	0.196
Breast meat	MDA	10.6 ± 2.62^a^	8.6 ± 0.88^a^	7.7 ± 0.96^ab^	8.0 ± 1.04^b^	1.87	0.131
	SOD	47.2 ± 5.67^b^	52.8 ± 6.43^ab^	63.0 ± 8.00^a^	62.7 ± 6.31^a^	9.25	0.068
	GSH-Px	0.7 ± 0.10^b^	0.9 ± 0.13^ab^	1.0 ± 0.15^a^	1.0 ± 0.20^a^	0.20	0.332

All measurements were expressed as Mean ± SD (*n* = 6). ^a,b^Means with different letters in the same rows are different significantly (*P* < 0.05). Birds supplemented with basal diet (CON), supplemented with 20 mg bacitracin/kg (ANT), supplemented with 50 mg BLFs/kg (BLFL) or supplemented with 250 mg BLFs/kg (BLFH)

### Effects of BLFs on the untargeted metabolomes of broilers’ breast meat

**Fig. 2:**
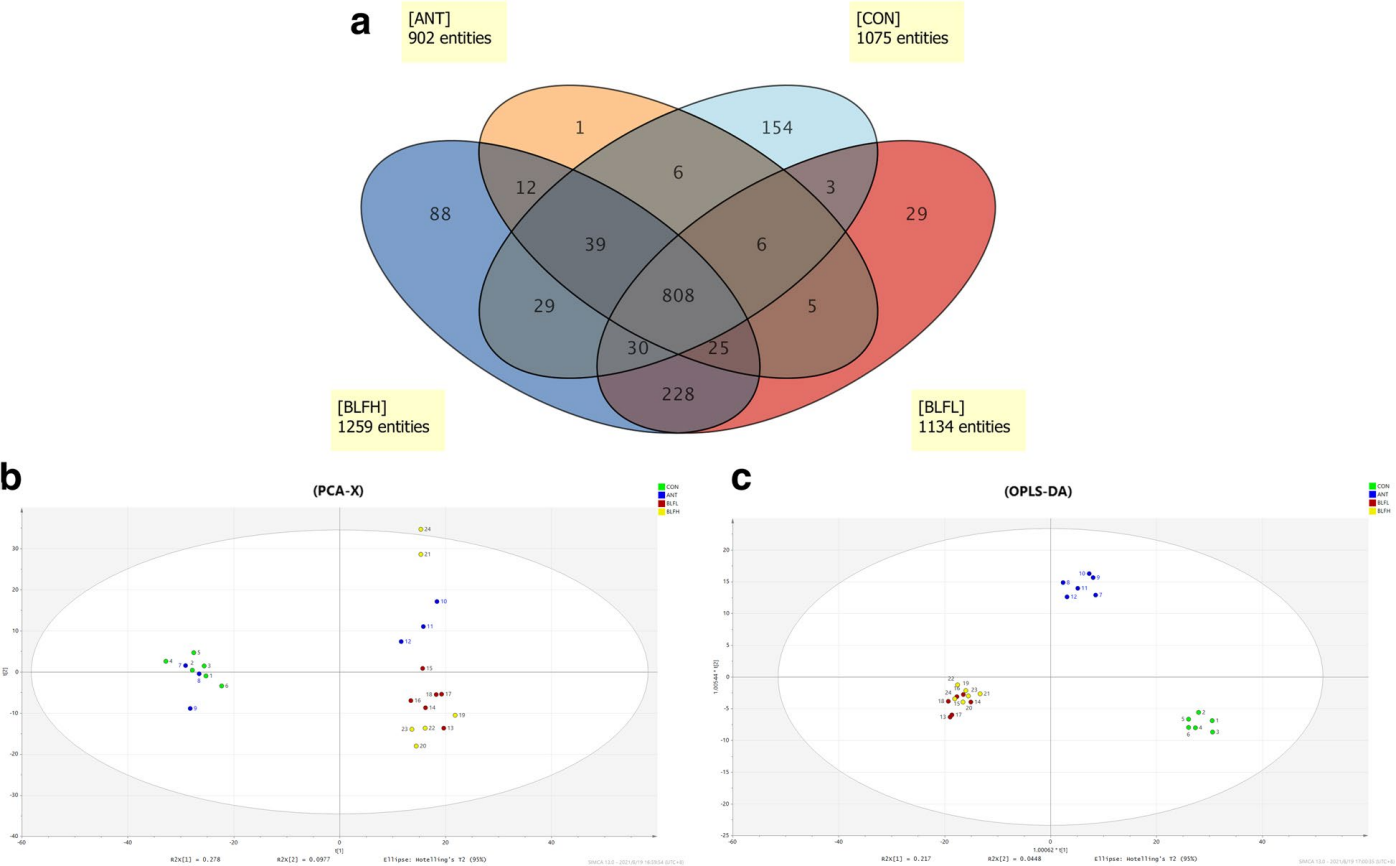
Effects of BLFs on untargeted metabolome of broilers’ breast meat. Note: Panel **a**, **b** and **c** represent Venn diagram, PCA plots and OPLS-DA plots analysis (*n* = 6). Birds supplemented with basal diet (CON), supplemented with 20 mg bacitracin/kg (ANT), supplemented with 50 mg BLFs/kg (BLFL) or supplemented with 250 mg BLFs/kg (BLFH)

The Venn diagram shows the 808 metabolites identified in all experimental groups, whereas 88 and 29 unique metabolites were found in the BLFH and BLFL groups, respectively (Fig. 2a), which the unique metabolites were increased along with the increasement of BLFs. The distribution of the principal component analysis scores in the BLFH and BLFL groups was significantly different from that in the CON group. The samples from the BLFH and BLFL groups were mainly concentrated in the fourth quadrant; those from the CON group were mainly concentrated in the second quadrant; and those from the ANT group were mainly concentrated in the first quadrant. In addition, the BLFL group showed proper aggregation, whereas the CON and ANT groups showed significant outliers (Fig. 2b). Meanwhile, orthogonal partial least squares discriminant analysis showed significant differences between BLF treatments (BLFL and BLFH groups) and control treatments (CON and ANT groups).

The contents of pumiloside, citranaxanthin (flavonoids), amino acids (N-gamma-glutamyl-s-propylcysteine, 2-amino-isobutyric acid, and isoleucyl-valine), and organic acids (cinnavalininate and 3-hydroxy-3-methyl-glutaric acid) in the BLF groups were significantly higher than those in the CON group (Table 11). In addition, the levels of the phenolic compound kanzonol W, aromatic compound 3-*p*-coumaroyl-1,5-quinolactone, and biliverdin IX were also significantly upregulated by BLF treatment.

**Table 11:** Comparison of the transportation of fourteen metabolites across the broilers’ breast meat

No	Compounds	Formula	Related category	RT, min	Mass (*m/z*)	BLFL vs. CON		BLFH vs. CON		ANT vs. CON	
						Trend	*P*-value	Trend	*P*-value	Trend	*P*-value
1	Pumiloside	C_26_H_28_N_2_O_9_	Alkaloids	20.407	572.2	Up	0.000	Up	0.000	Up	0.007
2	Psychosine sulfate	C_24_H_47_NO_10_S	Glycosides	13.734	601.3159	Up	0.000	Up	0.000	Up	0.006
3	N-gamma-Glutamyl-S-propylcysteine	C_11_H_20_N2O_5_S	Amino acid	13.032	352.1322	Up	0.001	Up	0.000	Up	0.047
4	Isoleucyl-valine	C_11_H_22_N_2_O_3_	Amino acid	4.488	230.1627	Up	0.000	Up	0.000	Up	0.006
5	2-amino-isobutyric acid	C_4_H_9_NO_2_	Amino acid	0.758	103.0632	Up	0.000	Up	0.000	Up	0.006
6	1-Methylhistidine	C_7_H_11_N_3_O_2_	Amino acid	0.749	169.0848	Up	0.000	/	0.000	Up	0.007
7	Kanzonol W	C_20_H_16_O_5_	Phenolic compounds	10.935	336.1018	Up	0.000	Up	0.002	Up	0.016
8	Citranaxanthin	C_33_H_44_O	Flavonoid	20.591	516.3593	Up	0.000	Up	0.000	Up	0.023
9	Cinnavalininate	C_14_H_8_N_2_O_6_	Organic acid	0.739	300.0403	Up	0.000	Up	0.000	up	0.006
10	3-Hydroxy-3-methyl-glutaric acid	C_6_H_10_O_5_	Organic acid	0.744	162.0526	Up	0.010	Up	0.017	Up	0.042
11	(+)-Chebulic acid	C_14_H_12_O_11_	Organic acid	5.291	356.039	Up	0.000	/	0.000	Up	0.006
12	3-*p*-Coumaroyl-1,5- quinolactone	C_16_H_16_O_7_	Others (Aromatic compounds)	7.871	412.1006	Up	0.020	Up	0.020	Up	0.037
13	Biliverdin IX	C_33_H_34_N_4_O_6_	Others	11.638	582.2478	Up	0.028	Up	0.000	Up	/
14	4-Acetamido-2-aminobutanoic acid	C_6_H_12_N_2_O_3_	Others	0.775	160.0842	Up	0.000	Up	0.000	Up	0.006

Up represents the distinguished metabolites was up-regulated compared to the control group. ''/'' represents no statistical difference compared to the control group (*n* = 6). Birds supplemented with basal diet (CON), supplemented with 20 mg bacitracin/kg (ANT), supplemented with 50 mg BLFs/kg (BLFL) or supplemented with 250 mg BLFs/kg (BLFH)

### Correlations between texture profile, protein secondary structure, and changed metabolites of broilers’ breast meat

**Fig. 3:**
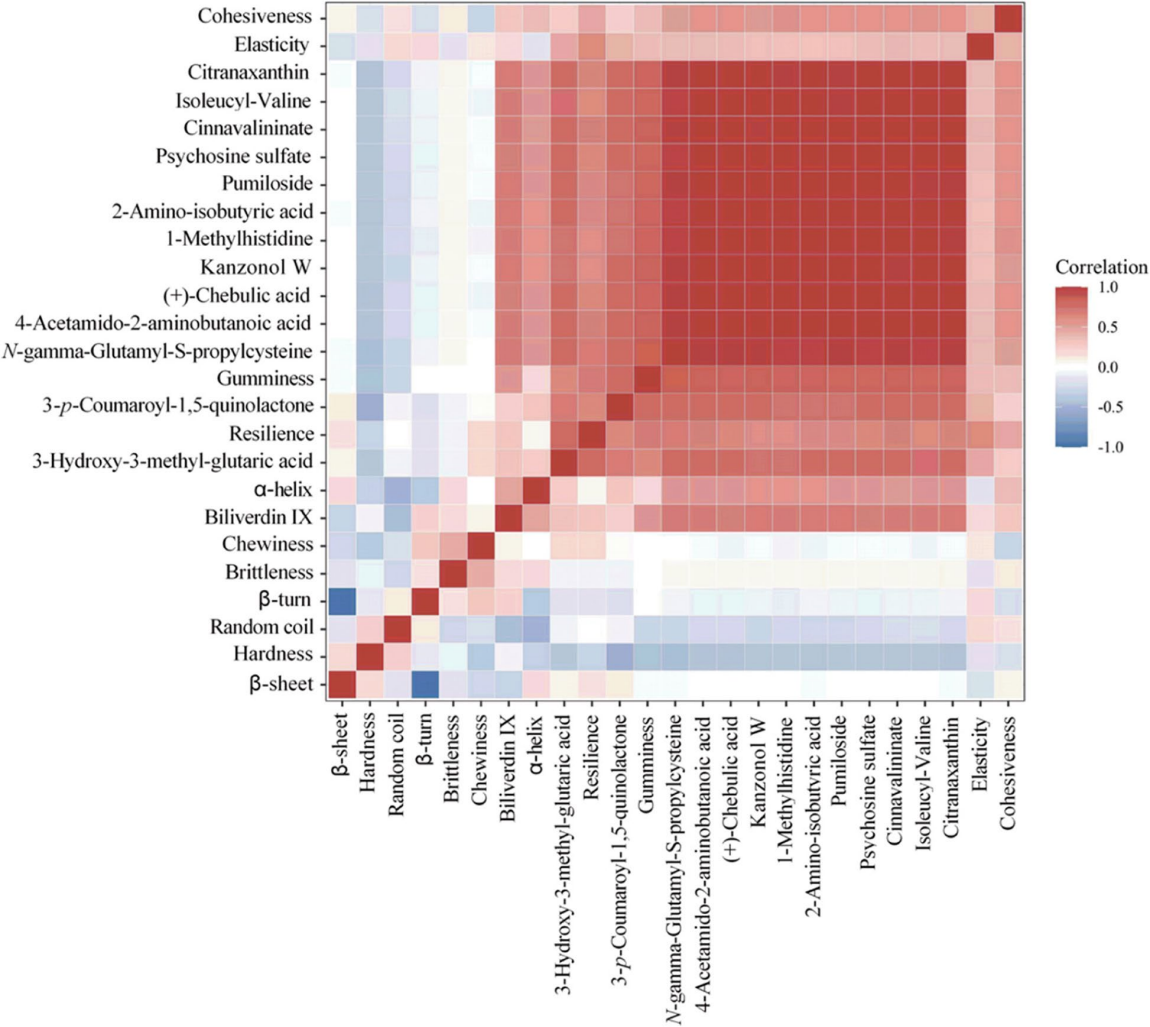
Correlations between texture profile, protein secondary structure and changed metabolites of broilers’ breast meat

Correlation heatmap analysis was performed to investigate the relationship between texture profile, protein secondary structure, and changed metabolites of broilers’ breast meat (Fig. 3). Metabolites that had changed significantly, except biliverdin IX, 3-hydroxy-3-methyl-glutaric acid, and 3-*p*-coumaroyl-1,5-quinolactone, were positively correlated with resilience, gumminess, cohesiveness, and α-helix, showing a correlation index value of approximately 0.3 to 0.7. However, a remarkable negative correlation was observed between hardness and significant altered metabolites except biliverdin IX, for which the value of the correlation index was lower than –0.5. In addition, the random coil of protein secondary structure was also negatively correlated with the significantly changed metabolites except for 3-hydroxy-3-methyl-glutaric acid, and 3-*p*-coumaroyl-1,5-quinolactone, showing a correlation index value of –0.2 to –0.6.

## Discussion

The present results were in accordance with the previous study [19], that is BLFs supplementation improved the ADG of broilers from d 21 to 42. Our previous study also found that BLFs treatment significantly enhances the ADG of birds from d 15 to 21 and d 1 to 21 [23]. The components and palatability of BLFs could improve the antioxidant function and growth performance of broilers [25]. Although our study found that the administration of BLFs did not influence the average daily feed intake of birds, the feed:gain ratio was significantly decreased compared to that of the control birds. It is possible that the dietary bamboo-derived flavonoids act as a type of growth hormone and promote the growth performance of livestock [26].

Studies have confirmed that two main groups of polyphenols, C-glycoside flavonoids and phenolic acids, play a role in the biological activities of bamboo leaves [22, 27]. Dietary supplementation with flavonoids has a great potential in improving the nutritional, sensorial, and microbiological qualities of poultry meat [28]. Fumalic acid supplementation has been found to alleviate the harmful effects of heat stress on growth performance and partial parameters of breast meat quality in broilers [19]. Data in the present study were also in accordance with previous results [19], in which dietary BLFH administration significantly increased the percentage of leg meat in broilers and significantly increased the breast percentage. The effects of flavonoid-rich plant extracts and potential mechanisms by which flavonoids enhance poultry production, including antioxidant, enzyme, and estrogenic activities have been investigated in a previous study [16].

Furthermore, texture profile indices indicate that shear energy, gumminess, and springiness positively correlate with firmness and the number of chews, whereas they negatively correlate with meat juiciness [29]. Vişan et al. found that beef marinated with vanilla and pressed oil has relatively lower levels of hardness, chewiness, and stickiness but has higher elasticity and cohesion [30]. The changes in collagen and texture properties of connective tissues may be attributed to the ability of organic acids to soften meat [31]. The present study showed that BLFs supplementation changed the meat texture profile of broilers by increasing the chewiness and gumminess, decreasing the cohesiveness of leg meat, and improving the elasticity of breast meat. In fact, supplementation with dietary flavonoid-rich plants and/or their extracts has gained increased interest in enhancing the production and meat quality of livestock [17]. However, no study has investigated the influence of dietary BLFs on the texture profile of broiler meat. Hence, further studies should be conducted to provide more evidence regarding the application of BLFs to alter broiler meat texture profiles.

Moreover, flavonoids reportedly confer their beneficial effects on water holding capacity by protecting the muscle tissue against stress-induced oxidative damage in broilers [17]. Bamboo leaf extract supplementation significantly increases drip loss at 24 h and pH at 45 min postmortem in broiler breast meat [19]. Similarly, treatment with purified flavonoids from enriched plants and their extracts significantly increase the water-holding capacity of breast meat [17]. However, there was no remarkable difference in the pH of meat among the treatment groups in the present study. Additionally, the numerical increase in Aw of leg meat was induced by the administration of BLFs, which was similar to the results of the abovementioned studies.

Furthermore, there are limited influences on carcass yield and texture profile; however, some evidence indicates that the normal physical meat quality indices change with the supplementation of plant-derived flavonoids, such as color, pH, and water-holding capacity. Kamboh et al. found that various plant flavonoids improve meat lightness by up to 5% [28]. Supplementation with flavonoid-enriched plants and their extracts lead to a remarkable increase in meat color (L* score) and pH in broilers [17]. Hu et al. found that broccoli stem and leaf meal (enriched with quercetin and xanthophylls) improve the shank a* value, decrease L* values, and improve the b* value in the shank and breast skin [32]. Similar to the above trials, we found that BLF supplementation significantly increased a* and c* values of breast meat and c* values of leg meat in broilers.

Intramuscular fat and connective tissues are closely related to meat tenderness, whereas the addition of sea buckthorn fruit flavanone increases intramuscular fat and influences fat deposition in broilers [33–35]. Previous studies have shown that flavonoids and polyphenols affect meat quality by affecting muscular shear energy, water-loss rate, fat distribution, and fiber properties in animals such as cattle and ducks [36–38]. Similarly, our study found that addition of BLFs decreased connective issues in breast muscle. In addition with the abovementioned effect of BLFs on conventional meat quality, the results of this study indicate that the bamboo leaf flavonoids used in this experiment had positive effects on improving broiler meat quality.

Few studies have determined the impact of dietary BLFs on protein structure. However, reports have confirmed that phenolic compounds can modify protein interactions by treating them as anti- or pro-oxidants [39]. The polyphenol compound caffeic acid induces the transformation of α-helix to β-fold in myofibrin, resulting in the strong antioxidant activity of the protein [40]. Similarly, our results indicated that BLFs supplementation induced a higher ratio of β-sheets and β-turns in comparison with the CON treatment. Huang et al. also confirmed that phenols promoted the unfolding of beef protein structure and the transformation from an α-helix to a β-turn structure [41]. Moreover, considering the undesirable oxidative changes in muscle proteins, polyphenols have beneficial effects on muscle proteins under oxidative processes [42, 43]. Pre-extracted plant phenols can induce protein unfolding and cross-linking of muscle proteins directly through reversible and irreversible bonds [39]. Hence, the chemical structure and concentration of flavonoids may be the major factors leading to changes in the protein structure.

Studies have demonstrated that bioflavonoids, considered a kind of natural antioxidant, could influence the antioxidative capacity, such as SOD and GSH-Px [44, 45]. BLFs can improve cell viability and reduce MDA content [46]. Our previous study confirmed that BLFs improve the serum antioxidative capacity of broilers at an early growth stage [23]. Consistent with the abovementioned study findings, BLFs supplementation in this study improved serum and breast antioxidative capacity of broilers, which mainly changed the meat texture profile.

To date, no trial has been conducted to study the influence of dietary BLF on the metabolome of broiler meat. In our study, BLF treatment significantly increased the levels of organic and amino acids. Furthermore, the levels of kanzonol W, 3-*p*-coumaroyl-1,5-quinolactone, and biliverdin IX were also significantly upregulated by BLF treatment. Li et al. showed that supplementation with eucalyptus polyphenol improves the amino acid composition of the breast muscle, especially histidine and glycine, in broilers [47]. According to a previous study, glutamic acid, *L*-isoleucine, and valerian are considered as flavor components [48]. Gamma-glutamyl-S-propylcysteine inhibits cholesterol synthesis [49]. Cholesterol and amino acids are closely related to meat quality and flavor [50]. These results confirmed that BLF could affect the deposition of the flavor-related substances and improve meat quality. In the absence of more similar historical evidence and more accurate data, further studies are needed to confirm the meat improvement effects of BLFs in poultry production.

## Conclusions

The administration of BLFH led to higher ADG and carcass yield, especially the leg and breast percentages of broilers. The higher levels of BLFs enhanced the antioxidative capacity and the texture profile of broiler meat, including increased chewiness, elasticity, and gumminess. The BLF supplementation resulted in a significantly increased c* value of meat sensory color, fewer β-sheets, and a higher β-turn ratio of protein secondary structure in the breast meat. Combined with the 14 significantly changed metabolites of breast meat, including flavonoids, amino acids, organic acids, and phenolic and aromatic compounds, as well as the correlations with the above parameters. The dietary BLF treatment can improve meat quality, and that changing protein secondary structures is associated with changes in antioxidative capacity and meat metabolites.

## Data Availability

The datasets used and/or analysed during the current study are available from the corresponding author on reasonable request.

## Abbreviations

ANT: Antibiotics
Aw: Water activity
BLFH: High bamboo leaf flavonoids
BLFL: Low bamboo leaf flavonoids
BLFs: Bamboo leaf flavonoids
CON: Control
GSH-Px: Glutathione peroxidase
MDA: Malondialdehyde
SOD: Superoxide dismutase
T-AOC: Total antioxidant capacity
